# Influence of Flunixin on the Disposition Kinetic of Cefepime in Goats

**DOI:** 10.1155/2014/471517

**Published:** 2014-05-05

**Authors:** Mohamed El-Hewaity

**Affiliations:** Department of Pharmacology, Faculty of Veterinary Medicine, University of El-Sadat City, Minoufiya 32897, Egypt

## Abstract

The pharmacokinetic profile of cefepime (10 mg/kg b.w.) was studied following intravenous and intramuscular administration of cefepime alone and coadministered with flunixin (2.2 mg/kg b.w.) in goats. Cefepime concentrations in serum were determined by microbiological assay technique using *Escherichia coli* (MTCC 443) as test organism. Following intravenous injection of cefepime alone and in combination with flunixin, there are no significant changes in the pharmacokinetic parameters. Following intramuscular injection of cefepime alone and in combination with flunixin, the maximum serum concentration was significantly increased in flunixin coadministered group compared with cefepime alone. However, no significant changes were reported in other pharmacokinetic parameters. The result of *in vitro* protein binding study indicated that 15.62% of cefepime was bound to goat's serum protein. The mean bioavailability was 92.66% and 95.27% in cefepime alone and coadministered with flunixin, respectively. The results generated from the present study suggest that cefepime may be coadministered with flunixin without change in dose regimen. Cefepime may be given intramuscularly at 12 h intervals to combat susceptible bacterial infections.

## 1. Introduction

It is well documented that concurrently administered drugs may alter pharmacokinetics of one or both drugs and in therapeutics antibiotic and nonsteroidal anti-inflammatory drugs (NSAIDs) are used most frequently in multiple drug prescriptions. Cefepime is a semisynthetic broad spectrum fourth generation cephalosporin antibiotic with a modified zwitterionic structure that allows more favorable penetration into the bacterial cells and reduced susceptibility to *β*-lactamases [1]. Cefepime shows broad spectrum of activity which includes Gram-positive cocci, enteric Gram-negative bacilli, and* Pseudomonas aeruginosa*. It lacks activity against methicillin-resistant* Staphylococcus aureus*, enterococci,* Bacteroides fragilis,* and* Listeria monocytogenes* [2]. Flunixin is nonsteroidal anti-inflammatory drug inhibiting cyclooxygenase enzymes in the arachidonic acid cascade, thus blocking the formation of cyclooxygenase derived eicosanoid inflammatory mediators [3]. Due to its anti-inflammatory, analgesic, and antipyretic effects [4], flunixin is widely used in veterinary medicine to treat the musculoskeletal conditions, acute mastitis, endotoxemia, and calf pneumonia [5, 6]. The pharmacokinetics of cefepime administered as a single drug has been investigated in many animal species including goats [7, 8], calves [9–11], cow calves [12], and sheep [13]. However, there is no available information on the influence of flunixin on the disposition kinetic of cefepime in goats. But there is some literature available on the influence of other NSAIDs on pharmacokinetics of cefepime as the effect of ketoprofen on disposition kinetic of cefepime in cow calves [12] and sheep [14]. The aim of the study was to determine the disposition kinetic of cefepime in goats after a single intravenous and intramuscular administration. More to assess the effect of flunixin co-administration on the disposition kinetic of cefepime in goats.

## 2. Materials and Methods

### 2.1. Drugs and Chemicals

Cefepime hydrochloride powder (Onsime) was purchased from Sigmatec Pharmaceutical Industries Egypt. Flunixin meglumine (Megloxyine) was purchased from ADWIA Pharmaceuticals Company Egypt. Mueller-Hinton agar was purchased from Mast Group Ltd., Merseyside, UK.

### 2.2. Animals

Twelve clinically normal goats were used in this investigation. The body weight ranged from 24 to 32 kg. Animals were housed in hygienic stable and fed on Berseem clover (*Trifolium alexandrinum*) dry concentrate. Water was provided* ad libitum*. None of the animals were treated with antibiotics for one month prior to the trial. The experiment was performed in accordance with the guidelines set by the Ethical Committee of El-Sadat City University, Egypt.

#### 2.2.1. Experimental Design

Goats were randomly divided into two groups six goats each. The 1st group received cefepime 10 mg/kg b.w. as a single intravenous dose into the right jugular vein and single intramuscular dose into the deep gluteal muscle with 2-week washout period between each route. Those of the 2nd group were given a single dose of flunixin (2.2 mg/kg b.w. IM) followed immediately cefepime 10 mg/kg b.w. by intravenous and intramuscular routes with 2 weeks of washout period between each route. Blood samples were collected at 5, 10, 15, and 30 minutes and 1, 2, 4, 8, 12, 18, and 24 h after drug administration. Blood samples were left to clot for 1 hour at room temperature; the clear sera were separated by centrifugation at 3000 r.p.m for 15 minutes and stored at −20°C until assayed.

#### 2.2.2. Drug Bioassay

The concentration of cefepime in serum samples was estimated by a standard microbiological assay method described by [15] using* Escherichia coli* (MTCC 443) as test organism [7]. This method estimated the level of drug having antibacterial activity, without differentiating between the parent drug and its active metabolites. The application of microbiological assay for measuring cefepime concentration is suitable [7]. Six wells were made at equal distances in standard Petri dishes containing 25 mL seeded agar. The wells were filled with 100 *μ*L of either the test samples or the cefepime standard concentrations. The plates were kept at room temperature for 2 h before being incubated at 37°C for 18 h. Zones of inhibition were measured using micrometers, and the cefepime concentrations in the test samples were calculated from the standard curve. Cefepime standard solution of concentrations from 0.195 to 50 *μ*g/mL. was prepared in antibiotic-free goat serum and phosphate buffer saline. Standard curves of cefepime were prepared in antibacterial-free goat serum by the appropriate serial dilution. The standard curve in goat serum was linear over the range of 0.195 to 50 *μ*g/mL and the value of correlation coefficient (*r*) was 0.991. The limit of quantification was 0.195 *μ*g/mL. Protein binding of cefepime was estimated according to [16].

### 2.3. Pharmacokinetic and Statistical Analysis

Following IV administration, the serum concentration versus time data of cefepime alone and coadministered with flunixin was fitted to a two-compartment open model system according to the following biexponential equation [17]:

(1) $$\begin{array}{c} C_{p}={Ae}^{-at}+Be^{-\beta t}, \end{array}$$

where *C*_*p*_ is the concentration of drug in the serum at time *t*, *A* and *B* are the zero-time drug intercepts of the distribution and elimination phase expressed as *μ*g mL^−1^, *α* and *β* are the distribution and elimination rate constants expressed in units of reciprocal time (h^−1^), and *e* is the natural logarithm base.

A pharmacokinetic computer program (R-strip, Micromath, Scientific software, USA) was used to determine the least-squares best-fit curve for cefepime concentration versus time data. Following IV and IM administrations, the appropriate pharmacokinetic model was determined by visual examination of individual concentration-time curves and by application of Akaike's information criterion (AIC) [18]. The pharmacokinetic parameters were reported as mean ± SE. Mean pharmacokinetic parameters after IV and IM administrations were statistically compared in cefepime alone and coadministered with flunixin using Student's *t*-test [19].

## 3. Results

**Figure 1:**
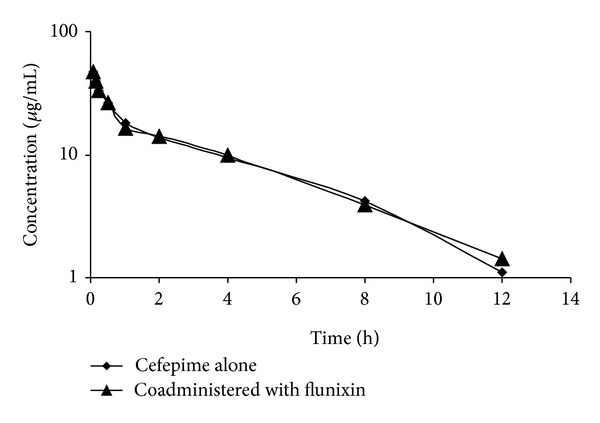
Serum concentrations of cefepime alone and in combination with flunixin following a single intravenous injection in goats.

**Figure 2:**
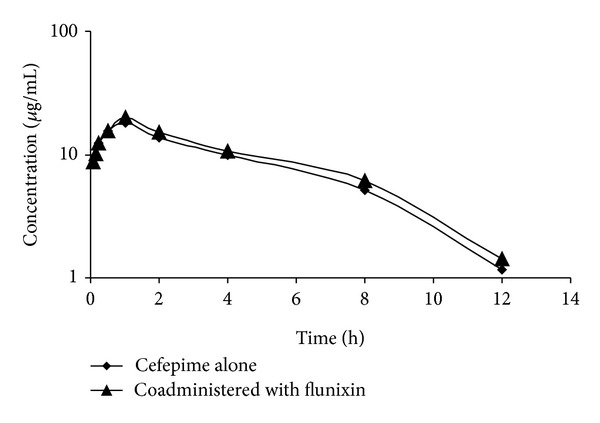
Serum concentrations of cefepime alone and in combination with flunixin following a single intramuscular injection in goats.

No clinical signs of adverse effects or intolerance were observed to cefepime after IV or IM injection. Mean serum concentrations of cefepime in goat following IV and IM injection of 10 mg/kg alone and coadministered with flunixin (2.2 mg/kg b.w.) are summarized in Figures 1 and 2. These data are best fitted to a two-compartment open model. The initial serum drug concentration following IV injection was 46.53 and 46.62 *μ*g/mL in cefepime alone and coadministered with flunixin, respectively, and was detected above MIC up to 12 h of administration in cefepime alone and coadministered with flunixin. Following IM injection of cefepime alone or coadministered with flunixin, the mean peak serum concentrations (*C*_max⁡_) were 16.49 ± 0.53 and 19.03 ± 0.71 *μ*g/mL achieved at time (*T*_max⁡_) 0.91 ± 0.08 and 1.01 ± 0.07 h, respectively. Cefepime could be detected in a therapeutic concentration for 12 h after IM injection in cefepime alone and coadministered with flunixin. The pharmacokinetic parameters of cefepime in goat following IV and IM injection of 10 mg/kg b.w. alone and coadministered with flunixin (2.2 mg/kg b.w.) are summarized in Tables 1 and 2. Following IV injection of cefepime alone and in combination with flunixin, there are no significant changes in the pharmacokinetic parameters. Following IM injection, the mean peak serum concentration (*C*_max⁡_) in goats was significantly increased in flunixin coadministered compared with cefepime alone. The result of* in vitro* protein binding study indicated that 15.62% of cefepime was bound to goat's serum protein. The mean bioavailability was 92.66% and 95.27% in cefepime alone and coadministered with flunixin, respectively.

## 4. Discussion

The pharmacokinetic of cefepime in goats is reported in the present study. The results revealed that serum cefepime concentration versus time decreased in a biexponential manner following IV injection either alone or when used concomitantly with flunixin, demonstrating the presence of distribution and elimination phases and justifying the use of two-compartment open model. This finding is in agreement with cefepime in goats [8]. Serum concentration showed a similar rapid distribution phase with elimination half-life of 3.34 and 3.50 h, respectively. This finding was similar to that recorded in calves 3.70 h [9] and cow calves 3.90 h [12]. Cefepime has moderate distribution in the body of goats with Vd_ss_ of 0.44 and 0.47 L/kg in cefepime alone and coadministered with flunixin, respectively. This Vd_ss_ was in agreement with that of the drug in cow calves 0.52 L/kg [12], in sheep 0.42 L/kg [13], and in calves 0.43 L/kg [9]. Mean value of the residence time (3.35 and 3.48 h) in cefepime alone and co-administered with flunixin, respectively. This finding was similar to that recorded in cow calves 3.38 h [10] and in calves 3.95 h [9], but longer than the value of 2.64 h recorded in goat [8]. Following intravenous injection of cefepime alone and in combination with flunixin, there are no significant changes in the pharmacokinetic parameters. These findings were similar to that recorded by [20] who found that there are no significant changes recorded in kinetic parameters of orbifloxacin (IV) when given with flunixin.

Following intramuscular administration of cefepime alone or coadministered with flunixin, no adverse effects or toxic manifestations were observed. The drug was very rapidly absorbed with a short absorption half-life *T*_1/2(ab)_ of 0.25 ± 0.02 h. The obtained result is consistent with those reported for cefepime in calves 0.21 ± 0.03 h [11]. The mean peak serum concentration (*C*_max⁡_) in goats was significantly increased in flunixin coadministered (19.03 ± 0.71 *μ*g/mL) compared with cefepime alone (16.49 ± 0.53 *μ*g/mL). A similar significant increase in peak serum level of cefepime following concomitant intramuscular administration of ketoprofen with cefepime has been observed in sheep [14]. A significant increase in peak serum level of ceftizoxime following concomitant intramuscular administration of paracetamol with ceftizoxime has been observed in cross-bred calves [21]. However, no significant alteration in *C*_max⁡_ was observed following coadministration of ketoprofen with cefepime in cow calves and coadministration of flunixin with orbifloxacin in buffalo calves [12, 20], respectively.

The *T*_1/2(el)_ was 3.44 ± 0.31 h which was shorter than cefepime in goat 4.89 ± 0.24 h [8], in sheep 5.17 ± 0.44 h [13], and in cow calves 5.15 ± 0.09 h [12]. The MRT was 4.01 ± 0.33 h which was similar to cefepime in goats 4.89 h [8], while being shorter than cefepime in sheep 6.89 h [13]. These differences are relatively common and are frequently related to interspecies variation, assay methods used, the amount of time between blood samplings and/or the health status, and age of the animal [22].

Following intramuscular administration of cefepime with flunixin in goat, none of the pharmacokinetic parameters were altered significantly (except *C*_max⁡_) in comparison to cefepime alone. Similarly there was no significant alteration in pharmacokinetic parameters (except *C*_max⁡_ and *T*_1/2(*α*)_) following coadministration of ketoprofen with cefepime in sheep [14], also the kinetic behavior of marbofloxacin in buffaloes was influenced by the coadministration with flunixin, and the affected parameters were *C*_max⁡_ and MRT [23] which support the results of our study. However, there are no significant changes in all pharmacokinetic parameters recorded by [20] who found that there are no significant changes recorded in kinetic parameters of orbifloxacin when given with flunixin in buffalo calves. Also, there is no significant change have been recorded in kinetic parameters of cefepime when given with ketoprofen in cow calves [12]. Variations in the pharmacokinetics of cefepime and other cephalosporins when given with NSAIDs have been observed in many experiments that may be due to differences in the chemistry of drugs and species difference.

Average serum concentration of 0.004–1.0 *μ*g/mL had been reported to be minimum inhibitory concentration (MIC_90_) for cephalosporins with various pathogens [24]. An average MIC_90_ of 0.5 *μ*g/mL of cefepime has been taken into consideration for calculation of efficacy predictors. Following intramuscular administration of cefepime alone or coadministered with flunixin in goats would result in a *C*_max⁡_/MIC_90_ ratio of 32.98- and 38.06-fold, respectively, which exceeds the recommended ratio of 10 and leads to potential clinical and bacteriological efficacy of cefepime [25, 26]. It is now accepted that high *C*_max⁡_/MIC_90_ values are necessary in order to avoid the emergence of bacterial resistance [27].

Based on this data, the intravenous and intramuscular injection of cefepime at dose of 10 mg/kg b.w. at 12 h interval in goat is sufficient to maintain serum concentration above MIC for most sensitive susceptible pathogens. The systemic bioavailability of cefepime in goats after IM administration alone and in combination with flunixin was 92.66 and 95.27%, respectively, which indicates excellent absorption of the drug. This finding was similar to that recorded in calves 98 ± 3% [11] and goats 86.45 ± 17.39% [7].

## 5. Conclusion

Cefepime can be used safely and effectively with flunixin for treating the infections and combating inflammatory conditions without alteration of the dose and dose intervals in goats. Further investigation should be done in the future to assess the effect of cefepime on the disposition kinetic of flunixin in goats.

## Figures and Tables

**Table 1:** Mean (±SE) kinetic parameters of cefepime (10 mg/kg b.w.) alone and in combination with flunixin (2.2 mg/kg b.w.) following a single intravenous injection in goats (*n* = 6).

Parameter	Units	Cefepime alone	Cefepime + flunixin
*T*_1/2(*α*)_	h	0.20 ± 0.004	0.20 ± 0.003
Vc	L kg^−1^	0.18 ± 0.007	0.18 ± 0.006
Vd_(area)_	L kg^−1^	0.46 ± 0.02	0.48 ± 0.03
Vd_ss_	L kg^−1^	0.44 ± 0.01	0.47 ± 0.04
*K*_12_	h^−1^	2.17 ± 0.04	2.26 ± 0.09
*K*_21_	h^−1^	1.47 ± 0.09	1.40 ± 0.04
*K*_el_	h^−1^	0.49 ± 0.02	0.50 ± 0.03
*T*_1/2(*β*)_	h	3.34 ± 0.12	3.50 ± 0.23
AUC_(0-inf)_	*μ*g mL^−1^ h^−1^	102.38 ± 8.61	103.91 ± 10.08
MRT	h	3.35 ± 0.22	3.48 ± 0.24
Cl_B_	L kg^−1^ h^−1^	0.098 ± 0.0004	0.096 ± 0.0003

*T*_1/2(*α*)_: distribution half-life; Vc: apparent volume of central compartment; Vd_(area)_: apparent volume of distribution calculated by area method; Vd_ss_: volume of distribution at steady state; *K*_12_: first-order constant for transfer from central to peripheral compartment; *K*_21_: first-order constant for transfer from peripheral to central compartment; *K*_el_: elimination rate constant; *T*_1/2(*β*)_: elimination half-life; AUC_(0-inf)_: area under serum concentration-time curve; MRT: mean residence time; Cl_B_: total body clearance.

**Table 2:** Mean (±SE) kinetic parameters of cefepime (10 mg/kg b.w.) alone and in combination with flunixin (2.2 mg/kg b.w.) following a single intramuscular injection in goats (*n* = 6).

Parameter	Units	Cefepime alone	Cefepime + flunixin
*T*_1/2(ab)_	h	0.25 ± 0.02	0.28 ± 0.03
*T*_1/2(el)_	h	3.44 ± 0.31	3.50 ± 0.22
*C*_max⁡_	*μ*g·mL^−1^	16.49 ± 0.53	19.03 ± 0.71*
*T*_max⁡_	h	0.91 ± 0.08	1.01 ± 0.07
AUC_(0-inf)_	*μ*g·h·mL^−1^	94.87 ± 3.89	98.99 ± 4.01
MRT	h	4.01 ± 0.33	4.08 ± 0.28

*P < 0.05 significant difference.

*T*_1/2(ab)_: absorption half-life; *T*_1/2(el)_: elimination half-life; *C*_max⁡_: maximum serum concentration; *T*_max⁡_: time to peak serum concentration; AUC_(0-inf)_: area under serum concentration-time curve; MRT: mean residence time.
